# Visuospatial representation in patients with mild cognitive impairment: Implication for rehabilitation

**DOI:** 10.1097/MD.0000000000031462

**Published:** 2022-11-04

**Authors:** Abiot Y. Derbie, Meseret A. Dejenie, Tsigie G. Zegeye

**Affiliations:** a Applied Cognitive Neuroscience Laboratory, Department of Rehabilitation Sciences, The Hong Kong Polytechnic University, Hong Kong; b Department of Psychology, Bahir Dar University, Bahir Dar, Ethiopia; c Department of Special Needs, Bahir Dar University, Bahir Dar, Ethiopia.

**Keywords:** allocentric spatial representation, egocentric spatial representation, Mild cognitive impairment, reference frames, visuospatial attention

## Abstract

Behavioral and neurophysiological experiments have demonstrated that distinct and common cognitive processes and associated neural substrates maintain allocentric and egocentric spatial representations. This review aimed to provide evidence from previous behavioral and neurophysiological studies on collating cognitive processes and associated neural substrates and linking them to the state of visuospatial representations in patients with mild cognitive impairment (MCI). Even though MCI patients showed impaired visuospatial attentional processing and working memory, previous neuropsychological experiments in MCI largely emphasized memory impairment and lacked substantiating evidence of whether memory impairment could be associated with how patients with MCI encode objects in space. The present review suggests that impaired memory capacity is linked to impaired allocentric representation in MCI patients. This review indicates that further research is needed to examine how the decline in visuospatial attentional resources during allocentric coding of space could be linked to working memory impairment.

## 1. Introduction

### 1.1. Visuospatial representation

On the busiest night in a cocktail party, you are attending your friend sitting in front of you, besides noises at the background (the voice of other partygoers, clinking of glasses, the background music, etc). You are trying to listen to every word that your friend is saying by lining your body forward and trying to ignore background noise at a time. While listening to your friend, someone from the other corner of the room mentions your name, and suddenly you become curious about what they are talking about. After a while, you asked your friend what s/he had said a moment earlier. Indeed, this scenario indicates that there is a shift in focused awareness and inquisitiveness to other environmental information, and this is one example illustrating how attention operates in daily life.

Our sensory organs constantly receive multiple billion bits of information per second. From our visual system alone, for example, we receive approximately 6 million bits of visual information per second.^[1]^ Humans rely more on visual information than other forms of sensory inputs.^[2,3]^ It is obvious that we cannot process all the visual stimuli that we are continuously presenting. Instead, we tend to attend to some stimuli and ignore others, as this would make the perception of the stimuli more effective and economical.^[4]^ The primary cognitive function related to such processes is attention, which is a gateway for higher-order cognitive processing.^[5]^

The American Psychological Association (APA) defines attention as “a state of focused awareness on a subset of the available perceptual information.” Posner et al developed an attention model consisting of 3 separate networks and integrated subsystems: alerting, orienting, and executive control.^[6,7]^ The alerting subsystem is important to show the vigilance of searching for general environmental stimuli directly through our sensory apparatus, such as being aware of other partygoers’ activities in a cocktail party scenario. The orienting subsystem diverts from the present consciousness to another new target (e.g., from his company to another individual sitting at a different corner in the party), shifting the focus to the present target (e.g., the individual at the corner), and engaging in the new target for further processing (e.g., listening to what the individual is saying).^[7]^ The executive subsystem is responsible for the recognition and identification of stimuli and responses and/or actions.^[6]^

**Figure 1.**
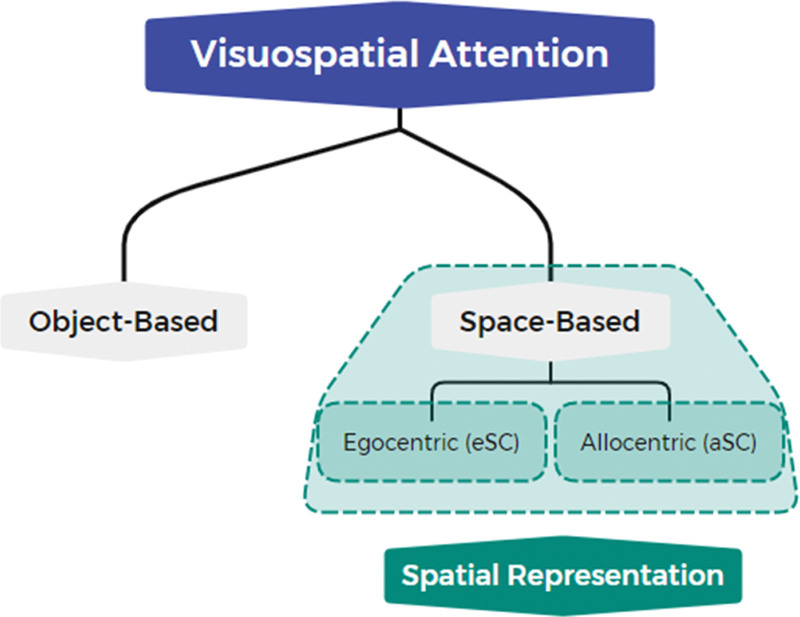
Classification of egocentric and allocentric representations of space.

One way to understand attention is to take an analogy, as information is coded and filtered before it reaches the brain for processing.^[8]^ Researchers have suggested that there are 2 types of filtering: space- and object-based.^[9,10]^ In object-based filtering, attention is allocated to the overall structure of object^[11]^ in space-based filtering; attention is allocated to space.^[11]^ Space-based filtering can be further divided into egocentric (objects are coded respective to the viewer coordinate frames) and allocentric (objects are coded according to the coordinates of other objects) representations^[12]^ (Fig. 1). Spatial representation is defined as the coordinate frame in which an individual is used to relate oneself to outside objects located in the environment.^[13,14]^

Egocentric representation refers to the position of the object encoded with reference to the observer coordinates, specifically some body parts, such as the leg.^[15]^ Allocentric representation, on the other hand, refers to the position of an object encoded relative to the spatial coordinates of other object(s), including different components of the object.^[16]^ Both allocentric (e.g., a ball at the center of the pitch) and egocentric (e.g., a ball at my right corner near the pitch) representations operate in different contexts in our visual system.^[17]^ Allocentric representation is sensitive to the overall properties of an object relative to its surroundings (e.g., object orientation, size, and color), whereas egocentric representation is independent of the contextual properties of the environment.^[17]^ De Wit et al reported that allocentric representations operate relatively slower than egocentric representations do.

In an egocentric representation, the relationship between a person and an object is crucial. Literature in this area shows that these reference coordinates begin in infancy.^[18]^ Allocentric representation, however, develops later in life and requires a deeper understanding of the spatial relationship between objects in space.^[19]^ It further requires the transformation of egocentrically coded information with, which adds to the relative spatial relationships among the objects.

From a developmental perspective, age-related differences in spatial representation have been reported between younger and older participants.^[13]^ Among children and young adults, objects were coded egocentrically.^[20]^

### 1.2. Neural substrates of allocentric and egocentric representations of space

#### 1.2.1. Overview of dorsal and ventral attention networks.

Attention function has been revealed to be associated with dynamic interplay among different neural fields, in other words, neural networks.^[21]^ Ungerleider^[22]^ proposed the dorsal pathway which is related to “where” property of objects; while the ventral pathway which is related to the “what” property of objects. The ventral stream, which is involved in visual object identification, comprises striate, prestriate, and inferior temporal areas. The dorsal stream, which is involved in the visual location of objects, comprises striate, prestriate, and inferior parietal areas.

Goodale and Milner^[23]^ proposed a different model for visual attention. There are 2 components: “visual for perception” and “visual for action.” According to them, visual perception and action are executed via 2 separate neural pathways. These 2 pathways start from the primary visual cortex and expand to the dorsal and ventral sides. The dorsal stream is used to identify objects (perception) and the ventral stream controls the visual field (action). Goodale and Milner’s model is useful in describing how visual attention is involved in encoding upcoming visual information. De Wit et al^[17]^ further postulated that the ventral stream requires the transformation of egocentric information so that the brain can encode the spatial relationships among objects in space. This calls for the features of objects and their prior relationships and associates them with the present object, which is likely to involve the memory system, and hence would take more time to process than the dorsal stream.

Recent studies have added to our understanding of distinctive dorsal and ventral attention pathways. The dorsal attention pathway mainly comprises the intraparietal sulcus and frontal eye field (FEF), whereas the ventral attention pathway involves the temporoparietal junction and ventral frontal cortex (VFC),^[24,25]^ with both initiating from the occipital cortex. The results of most of these studies were task-based, in which the participants acted in response to external demands. Fox et al^[26]^ demonstrated the involvement of similar neural substrates associated with the dorsal and ventral streams based on BOLD signals elicited from the spontaneous neural activities of participants without external demands.

Since Goodale and Milner,^[23]^ there have been further studies on enriching the neural mechanisms underlying egocentric and allocentric representations. Committeri, Galati^[27]^ and Zhang and Zheng^[28]^ demonstrated that frontoparietal networks, including the frontal eye-field, mediate egocentric representation. Egocentric representation is assumed to be limited to the dorsal visual pathways, whereas allocentric representation is relatively extended in both the dorsal and ventral visual pathways. Other studies have shown that egocentric and allocentric spatial representations mediate different anatomical areas and that their functions are dissociable.^[29,30]^ Inspired by Goodale and Milner^[23]^ and Vossel, Geng^[31]^ discussed the functional and anatomical area of attention in which the dorsal attentional networks start from the primary visual area to the FEF via the intraparietal sulcus. Likewise, Vossel et al^[31]^ showed that ventral attentional networks comprise the primary visual area to the FEF via the temporoparietal junction. The primary visual area is involved in both allocentric and egocentric coordinates.^[32]^ This assumption allowed us to ask whether there are cortical areas specialized in processing egocentric and allocentric coordinates in relation to the dorsal and ventral streams.

#### 1.2.2. Egocentric spatial representation.

Studies have suggested that the neural regions in the dorsal visual stream are egocentrically related to visual attention. To examine the role of the frontoparietal system related to the reference frame, Vallar, Lobel,^[30]^ and Derbie et al^[33]^ performed fMRI scans while subjects viewed a luminous vertical bar moving horizontally, and were required to press a button when the bar was straight ahead. The study concluded that the right posterior parietal and premotor regions are active during egocentric representation. Zaehle, Jordan^[32]^ also identified the precuneus as the key area for egocentric representation (for a recent neuroimaging meta-analysis, see ref.^[34]^).

Further evidence suggests that the superior parietal lobule also mediates egocentric representation in patients who show spatial neglect due to lesions in the parietal region. These patients manifest egocentric disorientation, which is an inability to locate the position of one’s own body parts.^[35,36]^ These findings suggest that the superior parietal lobule is important for encoding spatial representations of objects, regardless of one’s own body parts or external objects, using an egocentric reference frame (for a recent neuroimaging meta-analysis, see ref.^[34]^).

#### 1.2.3. Allocentric spatial representation.

In contrast to the egocentric reference frame, the allocentric representation is more strongly related to a different set of neural regions. Zaehle et al^[32]^ asserted that allocentric spatial representation, using verbal descriptions of spatial relationships, was able to activate a network comprising the right inferior parietal lobe and ventro-lateral-occipito-temporal cortex. Among patients with middle temporal gyrus and superior temporal gyrus lesions, allocentric neglect of the left side has been reported.^[37,38]^ These studies also noted that patients with right-hemisphere damage exhibited impaired egocentric representations. Committeri and Galati^[27]^ performed fMRI scanning by manipulating viewer-centered and object-centered tasks, and the subjects were asked to estimate the distance. They concluded that the ventral lateral occipital cortex was highly involved in allocentric spatial coordinates. Studies have demonstrated that the temporoparietal junction is the key region that mediates allocentric representation.

Neuropsychological and neurophysiological studies have attempted to correlate spatial neglect with spatial representation. Unilateral neglect studies have shown that both allocentric and egocentric representations might be affected differently. Ota et al^[39]^ reported 2 patients with brain injury due to stroke in different cortical areas. Both patients had right hemispheric damage and severe left neglect. Patient 1 had lesions in the superior temporal gyrus and inferior frontal gyrus, and patient 2 had lesions in the right parieto-tempororal area. The authors tested both allocentric and egocentric neglect of these patients simultaneously using new figure discriminative cancelation tasks. In this task, patients were asked to differentiate between horizontal lines. Interestingly, patients with lesions in the superior temporal gyrus and inferior frontal gyrus showed impairment in performing the task related to the use of an egocentric reference frame, while their performance in the allocentric task was normal. In contrast, patients with parieto-temporo-occipital lesions showed impairment when using the allocentric reference frame, but a normal performance when using an egocentric reference frame. These findings suggest that both reference frames may be related to different neural mechanisms.

In summary, a review of the brain imaging literature suggests 3 different views of the neural processes involved in the 2 spatial representations. The first view stipulates that the cognitive processes underlying these 2 spatial representations are different and are subserved by distinct neural substrates distributed along the dorsal and ventral frontoparietal attention streams.^[26,31,40]^ The ventral stream is involved in allocentric representation, while the dorsal stream is maintained in egocentric representation.^[41,42]^ Previous functional imaging studies have identified that activation of the posterior parietal cortex (PPC) (part of the dorsal stream) is associated with processing visual maps involving egocentric representation.^[27,43]^ This contrasts with activations in the medial temporal lobe (part of the ventral stream), which were found to be associated with maintaining visual representations involving allocentric representation. Additional evidence also comes from patients with hemi-spatial neglect: lesions in the IPL are associated with egocentric neglect^[44]^ and lesions in the MTL are associated with allocentric neglect.^[45]^

The second view is that the 2 spatial representation types are maintained by a unified neural framework. Byrne and Becker,^[46]^ for example, argued that encoding information from the visual field requires translation between allocentric and egocentric representations. Neural processes have been proposed to be mediated by the PPC^[47,48]^ and the retrosplenial cortex, as projected from the PPC.^[49–51]^ Findings in patients with brain damage shed some light on a unified view. Yue and Song,^[38]^ based on the classical gap detection task, concluded that allocentric neglect was consistently associated with egocentric neglect among patients with right-hemisphere damage. Subsequent studies on unilateral neglect further confirmed the non-differentiable view.^[37,52]^ A recent review of brain imaging studies offered a plausible explanation that the activities involved in the 2 spatial representations appeared to be confounded by the nature and cognitive resource demands of the spatial task (critical review:^[53]^). These activities are mediated by the middle temporal lobe and the neural substrates in the parietal lobe.^[42]^

The third view is that neural substrates mediating egocentric spatial representation are subsumed under those mediating allocentric but not the other way.^[32]^ Egocentric representationis primarily transitory and updates the representations of the object in space.^[20]^ Allocentric representation is more enduring which incorporates “cognitive map” into the representations.^[54]^ Allocentric representation, when compared with egocentric representation, involves additional information processing processes such as visual working memory^[55]^ and demands greater cognitive resources (for critical review:^[53,56]^). Additional neural processes were reported to be mediated by the parietal cortex, particularly the precuneus, on top of the frontoparietal attention network.^[14,32,53]^ Visual working memory and spatial representations are closely related cognitive domains that can be dissociated using computational cognitive modeling (e.g., refs. ^[57,58]^).

### 1.3. Mild cognitive impairment

This study focused on mild cognitive impairment (MCI), particularly how egocentric and allocentric reference frames would be distinctive among individuals with MCI. MCI refers to cognitive decline and inability to meet the expected cognitive function to one’s age and educational level, yet their daily functioning is not interrupted and cannot be diagnosed as dementia.^[59]^ MCI is considered a transition state to Alzheimer’s-type dementia.^[59,60]^ This transition is characterized by impairments in memory, attention, and executive control. The boundary between MCI and normal aging and Alzheimer’s disease (AD) is not well defined, and MCI shares some characteristics of aging and AD.^[61]^

There is no clear cutoff for the diagnostic criteria for MCI.^[62]^ Earlier, MCI was linked only to mild memory impairments. The definition was later refined by Petersen,^[63]^ who classified MCI into subtypes, which is now one of the most widely used definitions for MCI diagnosis. Petersen classified MCI into 4 subtypes, based on 2 factors. First, if memory is impaired, a person will be diagnosed with amnestic-MCI (a-MCI); otherwise, the person will be described as having non-amnestic MCI (na-MCI). Second, the classification is subdivided according to whether there are single or multiple additional cognitive domains that are impaired. If a person has a-MCI and is not impaired in any other cognitive domain, the person is diagnosed with as *a-MCI-single domain*; if memory is impaired and accompanied by impairment of any other cognitive domain, the person is classified with an *a-MCI-multiple domain*. On the other hand, if a single non-memory domain is impaired, patients have *non-amnestic-MCI-single domain* whereas patients with multiple non-memory domains are described as *non-amnestic-MCI-multiple domain.*

Different subtypes of MCI are related to the prognosis of different neurodegenerative diseases. Amnestic MCI is more likely to develop into AD, while multiple and non-memory single domain MCI develops into other forms of neurodegenerative diseases, including vascular dementia, aphasia, and Parkinson’s disease.^[61]^ There is also another broader cause-effect-based view of MCI: cognitive impairment as a result of other diseases, which reduces the blood supply to the brain (vascular MCI), and impairment as a result of neurodegeneration.^[59]^ Disregarding of subtypes and diagnostic criteria being used, previous studies consistently showed that networks of memory, attention and executive functions are disrupted (^[64]^: meta-analysis of studies).

Patients with multiple-domain MCI, aMCI, and single non-memory-MCI scored significantly lower than healthy subjects in the Baycrest attention test, which was designed to measure visual attention and visuospatial skills.^[65]^ Redel et al^[66]^ administered a partial report task to assess selective visual attention and supported the view that selective attention is affected at the earliest stage of a-MCI. This behavioral study provides empirical evidence that patients with MCI exhibit visual attention deficits.

#### 1.3.1. Functional and structural changes of the brain in MCI.

Physiological changes in the brains of healthy elderly individuals and patients with MCI have also been studied. In this regard, the volume of gray matter was associated with attentional networks in both pathways. By administering the trail making test, Sousa and Gomar^[67]^ revealed that orienting attention is one of the major problems associated with MCI, and this deficit was linked to a reduced thickness of the lateral temporal cortex. More recently, Granziera et al^[68]^ conducted a multi-contrast MRI study to investigate possible microstructural damage among patients with amnestic and non-amnestic MCI. The results showed that the myelin and cellular membrane proteins of MCI participants were reduced, which correlated with their cognitive performance. Other MCI studies in MCI shown functional disconnections in different brain areas among the participants. For instance, fMRI studies by Sun et al,^[69]^ Liu et al,^[70]^ and Yao et al^[71]^ showed abnormal topographical patterns of brain networks in patients with MCI and AD. Such abnormal patterns were dominant in the occipital and medial temporal lobes. Recently, Wei and Li^[72]^ compared different brain networks in patients with MCI and in elderly controls. In their EEG study, subjects performed spatial and visual tasks in which they were required to attend to the color rather than the shape of the stimulus. The results revealed significant declines infrontoparietal networks in patients with MCI.

Lei et al^[73]^ used a voxel-based morphometric method and concluded that among MCI participants, there was significant atrophy in the gray matter surrounding the prefrontal cortex, especially in the dorsolateral prefrontal cortex. Event-related fMRI studies shown that the dorsolateral prefrontal cortex is associated with shifts of attention in both human and monkey subjects.^[74]^ Studies, in all subtypes of MCI, have also shown that thickness of the middle temporal gyrus (bilaterally) and entorhinal cortex were reduced and linked to poor cognitive performance in memory, attention, and processing speed.^[67,75]^ These results suggest that the ventral visual pathway, which is important for object recognition, is a hotspot of neurodegeneration in MCI.

Neuroimaging studies have also suggested that cortical atrophy could be a preceding factor in MCI development. One of the most significant atrophy which directly predicts the conversion of MCI from normal aging is atrophies in the superior and middle temporal gyri,^[76]^ left entorhinal cortex, hippocampus, amygdala^[77]^ and right inferior frontal gyrus.^[75]^

Functional specialization of the brain has been well documented. In recent decades, studying task-related interactionsin distant brain areas^[78]^ has become one way to observe the integration (interaction) of brain regions. Changing conditions or manipulating tasks only brings about specific task-related neural areas and does not entail how interactions among specific brain regions are related.^[78,79]^ No attempts have been made to determine the functional interaction of brain regions that mediate both allocentric and egocentric reference frames.

#### 1.3.2. Spatial representation in MCI: neural mechanisms.

Studies have demonstrated that attention could have an extensive impact on visual perception in the primary visual cortex and dorsal and ventral visual perception networks. Although these networks are distinctive in nature and role, the 2 streams are assumed to be configured to produce visual perception.^[31]^ Currently, substantial clinical studies have demonstrated these two separate and integrated attention mechanisms.

The dorsal and ventral visual pathways of patients with MCI have been investigated in relation to Posner’s model of attention. The dorsal visual system (involving the intraparietal sulcus and FEFs) is assumed to mediate goal-directed attention.^[26,31]^ On the other hand, the ventral visual system comprises the temporoparietal junction and VFC, while controlling stimulus-driven (exogenous) orienting visual attention^[28,80,81]^ using both behavioral attention tests and event-related fMRI in patients with MCI and AD. They demonstrated that the dorsal attentional networks were functionally degenerated, while the ventricles were selectively deactivated in patients with aMCI.

Damage (or loss of volume) in the posterior parietal lobe (see Posner and Petersen, 1990) will lead patients with MCI to have difficulty orienting to visual stimuli. This further leads to impairment in egocentric representation. These should also be apparent in patients with progressive deterioration in the superior colliculus and/or surrounding areas, which also show a deficit in the ability to shift attention. This is because the computation involved in moving attention to a target is impaired. Studies^[82]^ have shown that visuospatial impairment was the earliest sign of MCI as a result of both attention and memory impairment.

Whether these subsets of attention systems fit the assumptions of 2 attentional networks (dorsal and ventral) has also been investigated in both patients with MCI and healthy controls. Among healthy controls, frontoparietal cortical and temporal parietal junction control alerting, while the left and right posterior lobe masters orienting attention and executive control were able to activate the anterior cingulate and right and left frontal areas.^[83]^ From the literature, it appears that appreciative brain regions in patients with MCI are degenerated in both the dorsal (ranging from the FEF to the intraparietal sulcus) and ventral stream (VFC to temporoparietal junctions).

Effective interaction between “vision for action and vision for perception” helps optimize the use of brain potentials. This may depend on the interaction of different neural areas in both the ventral and dorsal visual pathways. Based on the neurophysiological studies reviewed, we conclude that: allocentric and egocentric representations of space involve distinctive and neural areas. The egocentric representation of space is associated with activation of the dorsal network comprising the posterior and intraparietal sulcus, precuneus, and FEF. On the other hand, the allocentric representation of space is associated with the ventral network comprising the temporoparietal junction and VFC; the allocentric representation involves transformation of egocentrically encoded information into spatial relationships between 2 objects in space, implying that allocentric representation involves memory function in addition to those of the egocentric representation. Since memory function is impaired at the earliest stage in patients with aMCI, as shown in several studies, allocentric representation could be impaired compared to that in healthy younger and older participants. Individuals with aMCI may rely more on egocentric than allocentric representation; neural underpinnings mediating allocentric representation space (i.e., from the VFC to the temporoparietal junction) are implicated to be impaired among patients with MCI. This was exemplified in the form of atrophy and reduced activity in these neural regions; MCI patients have fewer functional interactions in those neural areas mediating both egocentric and allocentric representations.

#### 1.3.3. Implication for future study.

In both developed and developing nations, the fertility rate is decreasing, while life expectancy is moving upwards due to combined factors. China, for example, has experienced a record increment in life expectancy from 1960 to 2005 in the world.^[84]^ It is clear that the aging population in developed nations (e.g., Japan) and modern metropolitan cities (e.g., Hong Kong) is increasing. Neurophysiological studies have consistently demonstrated that aging is associated with various neurodegenerative diseases. For the past few decades, among neurodegenerative diseases, research has focused on MCI. Studies have shown that the prevalence of progression of MCI to dementia and other types of neurodegenerative disease ranges from 18.5% at the age of 50 years to 38.4% at the age of 78 years (e.g., see ref.^[85]^ for review). In the past few decades, has focused on the etiology, associated symptoms, prognosis, and behavioral outcomes; however, little is known about the neural mechanisms and dynamics of visual attention in patients with MCI. The lack of studies on the neural underpinnings of visual attention in patients with MCI has resulted in the following gaps:

1. Studies have shown that patients with MCI have difficulty with spatial navigation. Little is known about the neuropathological mechanisms underlying visual attention in patients with MCI, which could have been helpful in predicting who, among MCI patients, will progress to dementia by having clear biomarkers of how patients with MCI frame objects in space in their daily activities.
2. Previous studies in MCI have emphasized memory impairment, and their findings lack substantiating evidence on how memory impairment could be associated with spatial navigation and how patients with MCI coordinate frames could be explained at the neural level. Substantiating how memory impairment could influence attention or vice versa could have helped us to obtain a full picture of MCI. This is especially true in relation to allocentric representation. Previous studies have shown that allocentric representation of space is linked to memory function.
3. Neuropsychological classifications, such as the classification of MCI subtypes, could be refined by contemporary studies that uncover the neural mechanisms of how attention is mediated in patients with MCI. Neuroimaging findings could contribute to the identification of specific regions of interest as biomarkers for the pathogenesis of MCI. Previous studies have shown that specific brain regions mediate allocentric and egocentric representation of space in healthy adults, but have failed to unveil how neurodegeneration in these brain regions could potentially lead to the development of MCI.

Future studies in this area should be conducted to fill these gaps by examining the neural underpinnings of allocentric and egocentric representations of space in patients with MCI. The use of basic scales to decipher visual attention in MCI and/or other similar conditions in resource-limited settings^[86]^ and the use of mobile devices in diagnosis^[87]^ would be useful. Studies in this area would be useful in contributing to the optimization of neurological rehabilitation for MCI. Additionally, future studies should be able to determine whether visual attention, particularly allocentric and egocentric representations of space, is avaluable marker for identifying individuals with MCI. To this end, MCI patients’ representation of space should make use of functional and anatomical localization.

## Author contributions

AYD was involved in the designand concept development, data collection, data analysis, and drafting of the final manuscript. MAD was involved in collating the studies and drafting the manuscript. TGZ was involved in the collating studies and manuscript preparation. All authors have read and approved the final manuscript.

**Conceptualization:** Abiot Y. Derbie, Meseret A. Dejenie, Tsigie G. Zegeye.

**Validation:** Abiot Y. Derbie.

**Writing – original draft:** Abiot Y. Derbie.

**Writing – review & editing:** Abiot Y. Derbie, Meseret A. Dejenie, Tsigie G. Zegeye.
